# Novel Regulator MphX Represses Activation of Phenol Hydroxylase Genes Caused by a XylR/DmpR-Type Regulator MphR in *Acinetobacter calcoaceticus*

**DOI:** 10.1371/journal.pone.0017350

**Published:** 2011-03-24

**Authors:** Haiying Yu, Zixin Peng, Yuhua Zhan, Jin Wang, Yongliang Yan, Ming Chen, Wei Lu, Shuzhen Ping, Wei Zhang, Zhonglin Zhao, Shuying Li, Masahiro Takeo, Min Lin

**Affiliations:** 1 College of Biological Sciences, China Agricultural University, Beijing, China; 2 Key Laboratory of Crop Biotechnology, Biotechnology Research Institute, Chinese Academy of Agricultural Sciences, Ministry of Agriculture, Beijing, China; 3 Department of Materials Science and Chemistry, Graduate School of Engineering, University of Hyogo, Himeji, Hyogo, Japan; 4 National Centre for Plant Gene Research, Beijing, China; King's College London, United Kingdom

## Abstract

*Acinetobacter calcoaceticus* PHEA-2 utilizes phenol as its sole carbon and energy source and has a multi-component phenol hydroxylase-encoding gene operon (mphKLMNOP) for phenol degradation. Two additional genes, mphR and mphX, were found upstream and downstream of mphKLMNOP, respectively. The mphR gene encodes a XylR/DmpR-type regulator-like protein and is transcribed in the opposite direction to mphKLMNOP. The mphX gene is transcribed in the same direction as mphKLMNOP and encodes a protein with 293 amino acid residues showing weak identity with some unknown proteins encoded in the meta-cleavage pathway gene clusters for aromatic compound degradation. Disruption of mphR by homologous recombination resulted in the loss of phenol degradation while disruption of mphX caused significantly faster phenol degradation than in the wild type strain. Transcriptional assays for mphK, mphR, and mphX revealed that mphR activated mphKLMNOP transcription in the presence of phenol, but mphX partially repressed this activation. Gel mobility-shift assay demonstrated a direct interaction of MphR with the mphK promoter region. These results indicate the involvement of a novel repressor protein MphX in transcriptional regulation of phenol hydroxylase genes caused by a XylR/DmpR-type regulator MphR.

## Introduction

Besides being important industrially for the synthesis of chemical products such as resins, medicines, and dyes, phenol is also a major toxic contaminant in industrial wastewaters from oil refineries, petrochemical plants, and phenolic resin plants. Thus, efforts to improve the efficiency of wastewater treatment via phenol degradation have gained special attention. Such efforts have led to a deeper understanding of phenol biodegradation mechanisms [1], [2].

A large variety of soil bacteria are able to degrade aromatic compounds by employing different strategies for regulatory control at the level of gene expression. In aerobic phenol degradation by microorganisms, phenol is normally converted by phenol hydroxylase into catechol, which flows into an *ortho*-cleavage pathway or a *meta*-cleavage pathway after the cleavage of an aromatic-ring by catechol 1,2-dioxygenase or catechol 2,3-dioxygenase [2]. Phenol hydroxylase, catalyzing the initial reaction in phenol degradation, is a key rate-limiting enzyme. Many phenol hydroxylases from soil microorganisms can be classified into three groups: single- [3], [4], two- [5], [6], and multi- [7], [8], [9] component types. Microorganisms with multi-component phenol hydroxylase (mPH) are distributed worldwide and seem to be the most ubiquitous in the environment [10]. The mechanism of phenol oxidation by mPH and the localization, organization, expression, and regulation of the mPH-encoding genes have been investigated in order to gain a basic understanding of the mechanisms involved in phenol biodegradation [6], [7], [11], [12], [13], [14], [15], [16] and to develop practical applications, such as the treatment of soils contaminated with aromatic compound and the development of biosensors for the detection of such contaminants [13], [17]. The best-characterized example of the mPH-mediated pathway is the *dmp*/Dmp system of *Pseudomonas* sp. CF600 consisting of 15 *dmp* genes on the pVI150 plasmid [9], [18], where the upstream activator DmpR in the presence of aromatic effectors activates divergent transcription of the mPH operon (*dmpKLMNOP*) [18], [19]. This gene arrangement is highly conserved amongst several other phenol degradation gene clusters such as *mop*, *phc*, *aph*, *phl* and *phh* [7], [20], [21], [22], [23]. DmpR and its analogous regulators including XylR for toluene and xylene degradation [24], [25] form a large XylR/DmpR subclass in the NtrC protein family [26], [27]. The molecular mechanisms of *xyl*/Xyl and *dmp*/Dmp systems are well-characterized to be via activator binding to σ^54^-dependent promoter regions of respective target operons [14], [24], [25]. In addition to XylR/DmpR-type mediated regulation, co-regulation of mPH gene expression by different types of regulators has also been observed in *Comamona*s *testosteroni* strains TA441 and R5 [7], [23], [28], [29]. Furthermore, carbon catabolite repression and some host-dependent global regulations have been reported to affect the primary regulation by XylR/DmpR-type proteins [14], [30], [31].

In most cases, the catabolic pathway-specific regulators act as transcriptional activators of gene expression with few exceptions of GntR-type proteins, which are exclusively described as repressors. The use of repressors in the regulation of phenol degradation is not yet fully understood. We previously isolated phenol and benzoate degradation gene clusters spanning a 15.5-kb region from *Acinetobacter calcoaceticus* PHEA-2 [32], [33]. This region contains at least four putative transcriptional units, *mphKLMNOP*, *benM*, *benABCDE*, and *benKP*, in this order. The complete genome of *A. calcoaceticus* PHEA-2 was sequenced recently and has been deposited in GenBank under accession number CP002177. Establishment of the complete genome sequence enabled mapping of the entire catabolic gene cluster in the PHEA-2 chromosome. In this study, we compared organization of the phenol catabolic gene cluster in *A. calcoaceticus* PHEA-2 (*mph*) and with the other phenol degrading genes cluster. In addition, the result of the growth test on phenol and measurements of phenol-oxygenating activities suggested that phenol degradation in PHEA-2 was inducible and that *mphR* and *mphX* encoded an activator and a repressor, respectively, affecting expression of the mPH-encoding genes.

## Results

### Involvement of the *mph* genes in phenol degradation

**Figure 1:**
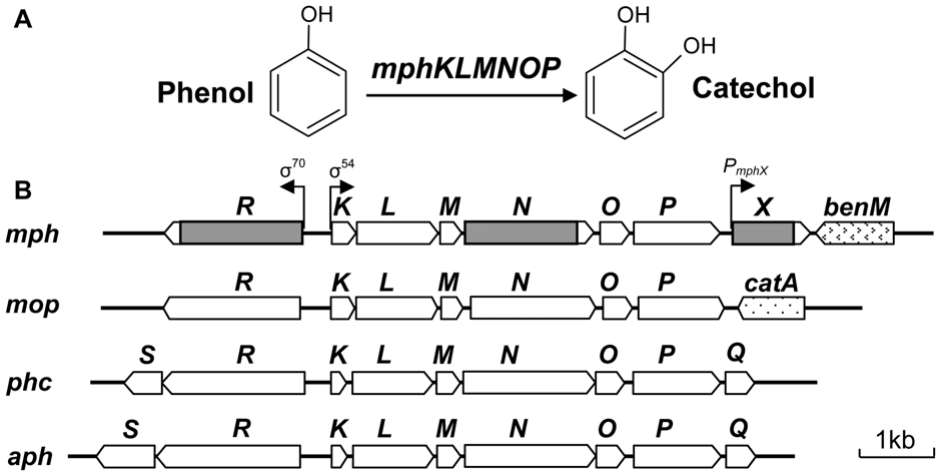
Phenol degradation in *A. calcoaceticus* PHEA-2. (A) Catabolic payhway for degradation of phenol to catechol. (B) Organization of the phenol catabolic gene cluster in *A. calcoaceticus* PHEA-2 (*mph*) and comparison with the other phenol degrading genes cluster in *A. calcoaceticus* NCIB8250 (*mop*), *C. testosteroni* TA441 (*aph*) and *C. testosteroni* R5 (*phc*). Genes are denoted by open arrows. The region of a deleted segment in *mphR*, *mphN* or *mphX*, respectively, is indicated by gray-shaded box.

**Figure 2:**
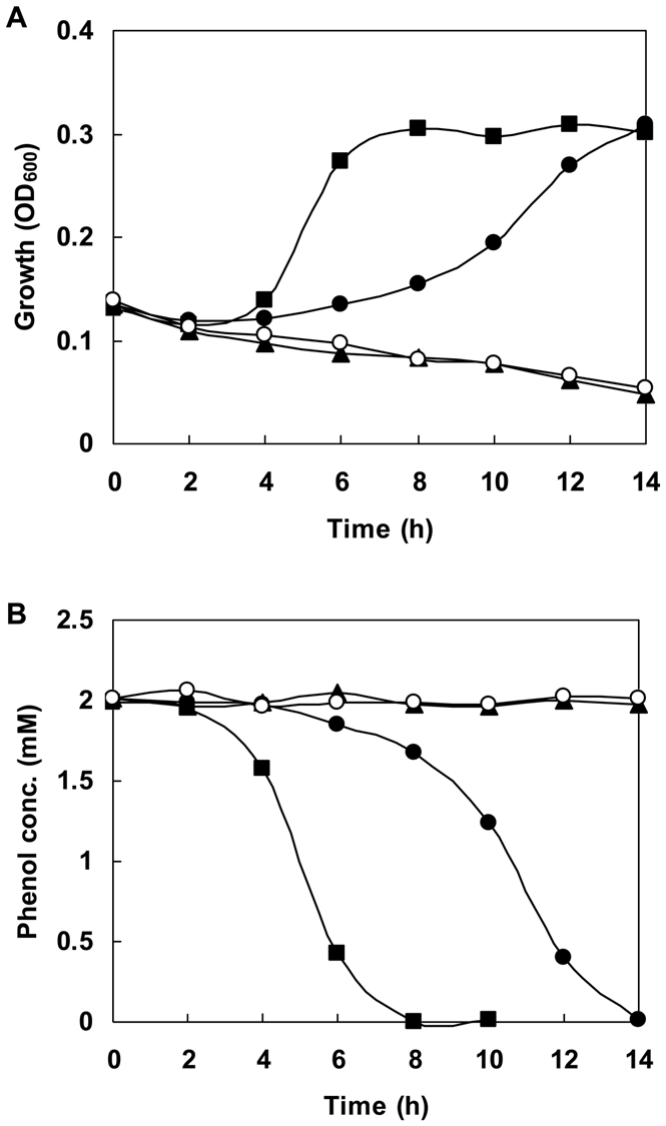
Growth and phenol degradation of *A. calcoaceticus* strains. Optical density (A) and phenol concentration (B) were monitored. Cells of *A. calcoaceticus* PHEA-2 (closed circles), *mphR*-deletion mutant A2R (closed triangles), *mphN*-deletion mutant A2N (open circles) and *mphX*-deletion mutant A2X (closed squares) were grown on 2 mM phenol in MS medium as the sole carbon source.

As described in the introduction, two gene candidates, *mphR and mphX*, were found upstream and downstream of the *mph* operon, respectively. From sequence analysis, *mphKLMNOP* was found to encode mPH proteins, catalyzing the initial reaction in phenol degradation (Fig. 1A). This multi-component enzymes exhibits 38–72% and 58–93% identity with proteins encoded by the cognate genes *dmpKLMNOP* of *Pseudomonas* sp. CF600 [9] and *mopKLMNOP* of *A. calcoaceticus* NCIB8250 [8], respectively. A *mphN*-deletion mutant lacking the center portion of the mPH-encoding operon (Fig. 1B) was constructed by homologous recombination and named A2N. It lost its phenol degradation activity (Fig. 2), but was still able to assimilate catechol (data not shown), showing that *mphKLMNOP* is essential for the initial step of phenol degradation in PHEA-2.

**Figure 3:**
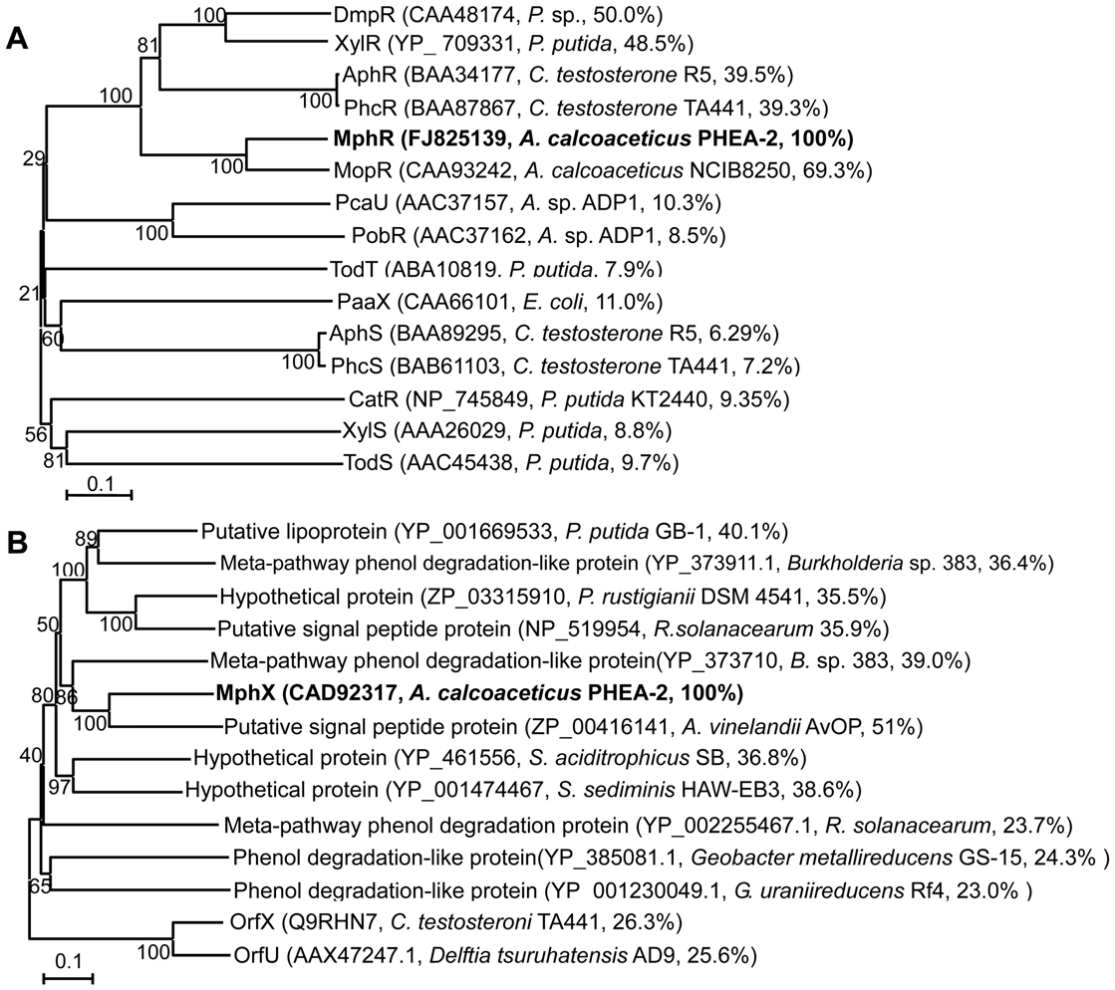
Phylogenetic tree from the alignment of the *A. Calcoaceticus* PHEA-2 MphR and MphX with related proteins. The condensed tree was obtained using Neighbor-Joining method of MEGA 4.0. The percentage of tree from 1000 bootstrap resamples supporting the topology is indicated when above 50. Accession numbers, strain names and sequence identity to MphR (A) and MphX (B), respectively, are shown in parentheses.

The MphR protein showed significant identity with XylR/DmpR-type regulators MopR (70% aa sequence identity), PhlR (52% identity), DmpR (51% identity), PhhR (51% identity), TouR (51% identity), CapR (51% identity) and XylR (48% identity), and expected to encode a XylR/DmpR-type regulatory protein homolog (Fig. 3A). To clarify the function of *mphR* in phenol degradation, a *mphR*-deletion mutant was constructed from PHEA-2 by homologous recombination and named A2R. The deletion was confirmed by PCR (the expected deleted segment is shown in Fig. 1B). As shown in Fig. 2, A2R was unable to grow on or degrade phenol at all. Introduction of the wild-type *mphR* into A2R using a complementation plasmid resulted in recovery of phenol degradation activity (data not shown). These results indicate that *mphR* encodes an activator for the expression of *mphKLMNOP* required for phenol degradation in PHEA-2.

In addition, we compared organization of the phenol catabolic gene cluster in *A. calcoaceticus* PHEA-2 (*mph*) and with the other phenol degrading genes cluster in *A. calcoaceticus* NCIB8250 (*mop*), *C. testosteroni* TA441 (*aph*) and *C. testosteroni* R5 (*phc*) (Fig. 1B). We found that an 882-bp open reading frame named *mphX* was detected between *mphKLMNOP* and *benM* and its gene product showed no significant similarity with any known functional proteins. To investigate the involvement of *mphX* in phenol degradation, an *mphX*-deletion mutant was constructed from PHEA-2 by homologous recombination and named A2X. Interestingly, *mphX*-deletion mutant A2X showed faster growth on phenol and faster phenol degradation than the wild-type PHEA-2 strain (Fig. 2). The *mphX*-complemented strain recovered the normal ability to grow on phenol with the growth rate similar to that of wild type (data not shown). In addition, in the phenol-oxygenating activity assay using cell suspensions, phenol-induced A2X cells showed higher activity (1.97±0.14 µmol/min/g-dry cell) compared to phenol-induced PHEA-2 cells (1.38±0.11 µmol/min/g-dry cell). However, lactate-grown cells of both strains showed similar basal level of activities (<0.05 µmol/min/g-dry cell). Therefore, it is possible that *mphX* encodes a repressor protein for the expression of the mPH genes. The MphX protein is comprised of 293 aa residues and is expected to have a molecular mass of 32.2 kDa. It shows weak identity with proteins encoded by *orfX* (27.8% aa sequence identity) in the phenol degradation gene cluster of *Comamonas testosteroni* TA441 [34] and *orfU* (26.3% identity) in the aniline degradation gene cluster of *Delftia tsuruhatensis* AD9 [35] whose functions are unknown. Searches found several homologous bacterial proteins with more than 30% identity, but the findings are annotated as putative or hypothetical proteins (Fig. 3B). MphX shows little identity (15% or less) with members of known regulator families (data not shown). Therefore, if MphX is a repressor, it can be classified as a new type of regulatory protein.

### Sequence analysis of the intergenic region between *mphR* and *mphK* and determination of the transcriptional start site for *mphK*

**Figure 4:**
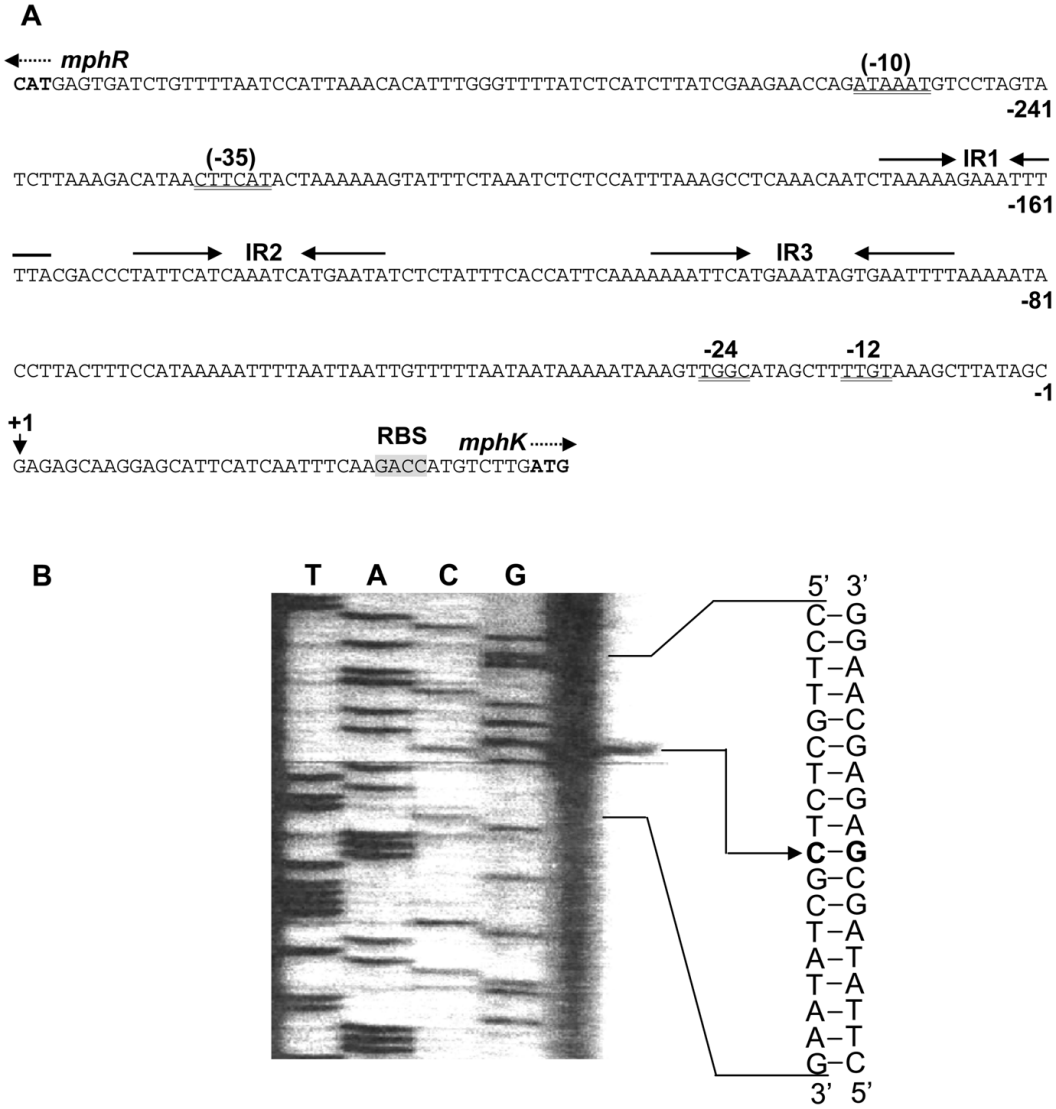
Analysis of the promoter region and transcriptional start site for mphK. (A) Intergenic region between *mphR* and *mphK*. The first nucleotide in the transcript is shown in bold. Arrows and dashed arrows indicate incomplete inverted repeats (named IR1, IR2, and IR3) and the translational start codons for *mphR* and *mphK*, respectively. Double-underlines mean the putative σ^54^-dependent −12/−24 promoter consensus sequence and the σ^70^-dependent −10/−35 promoter consensus sequences. An arrowhead with +1 indicates the transcriptional start site for *mphK*. A grey-shaded box indicates the putative ribosome-binding site for the *mphK* transcription. (B) Mapping of the transcriptional start site for *mphK* by primer extension. A sequence ladder was generated with the same primer (lanes T, A, C, and G).

As shown in Fig. 4A, in the intergenic region between *mphR* and *mphK*, three incomplete inverted repeat sequences (IR1, IR2, and IR3) [36], a long A/T-rich sequence (spanning from −134 to −29 relative to the putative transcriptional start site for *mphK*), and a putative σ^54^-dependent −12/−24 promoter sequence (5′-TTGGCATA-N_4_-TTGTA-3′, N = any base) for *mphK* were found. The putative promoter sequence is very similar to the consensus sequence of the −12/−24 promoters (5′-YTGGCACG-N_4_-TTGCW-3′, Y = C or T, W = A or T) described by Wigneshweraraj *et al.* [37]. To identify the transcriptional start site for *mphK*, primer extension analysis was performed using total RNA isolated from phenol-induced PHEA-2 cells. A single band corresponding to a G residue located 39 bp upstream from the translation initiation codon of *mphK* was detected (Fig. 4B). Considering the location of the −12/−24 promoter for *mphK*, this position seems suitable for the transcriptional start site for *mphK*.

In contrast, a putative σ^70^-dependent −10/−35 promoter sequence for *mphR* (5′-ATGAAG-N_23_-ATTTAT-3′) was also detected (Fig. 4A). This sequence is similar to the σ^70^-dependent promoter (5′-GTGAAG-N_19_-TAATAT-3′) of the *A. calcoaceticus* NCIB8250 *mopR* gene [22], suggesting that *mphR* is transcribed from the σ^70^-dependent promoter. The arrangement of *mphR-KLMNOP* and the above promoter/operator elements are nearly identical to those of several other phenol degradation gene clusters with a XylR/DmpR-type regulatory gene, such as *dmp*, *mop*, *phc*, *aph*, *phl* (strain H) and *phh* [7], [19], [20], [21], [22], [28].

### Regulation of the transcription of *mphKLMNOP*, *mphR*, *and mphX*

To evaluate the transcriptional activities of *P_mphK_*, *P_mphR_* and *P_mphX_* under various conditions, *P_mphK_-*, *P_mphR_*-, and *P_mphX_*-*lacZ* fusions were constructed in pGD926 and the resulting plasmids, pGDP, pGDRP and pGDPX, were independently introduced into the wild-type strain PHEA-2, the *mphR*-deletion mutant A2R and the *mphX*-deletion mutant A2X. After 6-h incubation with or without phenol, cell suspensions were prepared and β-galactosidase activity was measured as transcriptional activity using the cell suspensions. The result is summarized in Table 1. From the result, transcriptional regulation of these key *mph* genes can be explained as follows:

- Transcription of *mphK* (the *mphKLMNOP* operon): Phenol-induced PHEA-2 cells showed 10 times higher *P_mphK_* activity than that of uninduced PHEA-2 cells, indicating that the transcription of *mphK* was phenol-inducible in PHEA-2. Irrespective of induction conditions, however, *mphR* deletion resulted in complete loss of *P_mphK_* activity with or without phenol. This result demonstrated that *mphR* is essential for the transcription of *mphK* and encoded an activator protein. In contrast, the *P_mphK_* activity of phenol-induced A2X cells was 2.4 times higher than that of the phenol-induced PHEA-2 cells, suggesting that *mphX* partially represses the *mphR*-dependent activation in PHEA-2 cells.
- Transcription of *mphR*: Phenol-induced PHEA-2 cells showed 1.8 times higher *P_mphR_* activity than that of uninduced PHEA-2 cells. This indicates that the transcription of *mphR* was weakly inducible in the presence of phenol. However, since a considerable level of the *P_mphR_* activity was detected even in uninduced cells, a small amount of MphR is probably always produced even in the absence of effectors in PHEA-2 and may serve as a sensor for effectors coming from outside of the cells. Low *P_mphR_* activity observed in both phenol-induced and uninduced A2R cells suggests that MphR is also an activator for its own gene expression. Furthermore, the absence of *mphX* resulted in a 40% increase in *P_mphR_* activity (calculated from comparison of the *P_mphR_* activity in phenol-induced A2X cells with that in phenol-induced PHEA-2 cells), suggesting that *mphX* also represses the transcription of *mphR* in PHEA-2 cells. This result is in good agreement with the result of the measurement of the phenol-oxygenating activities shown above.
- Transcription of *mphX*: Phenol-induced of PHEA-2 cells showed a high level of the *P_mphX_* activity compared to that of uninduced PHEA-2 cells. However, in the absence of *mphR* or *mphX* (in A2R or in A2X), *P_mphX_* activities were at a basal level even under induction by phenol. These results indicate that the transcription of *mphX* was phenol-inducible and absolutely dependent on the presence of *mphR* and *mphX* itself. Very low *P_mphX_* activity in A2X cells even in the presence of *mphR* and phenol demonstrates that *mphX* might be in a transcriptional unit separate from *mphKLMONP*.

**Table 1:** β-Galactosidase activities of *mphK* promoter-, *mphR* promoter-, or *mphX* promoter-*lacZ* transcriptional fusion in *A. calcoaceticus* PHEA-2 and *mphR*- and *mphX*-deletion mutants (A2R and A2X).

	β-Galactosidase activity (Miller unit)^a^	
	+Phenol	−Phenol
PHEA-2(*P_mphK_-lacZ*)	336±33	34±2
A2R(*P_mphK_-lacZ*)	*	*
A2X(*P_mphK_-lacZ*)	807±58	30±2
PHEA-2(*P_mphR_-lacZ*)	160±20	92±10
A2R(*P_mphR_-lacZ*)	20±5	21±3
A2X(*P_mphR_-lacZ*)	225±33	176±15
PHEA-2(*P_mphX_-lacZ*)	332±50	*
A2R(*P_mphX_-lacZ*)	*	*
A2X(*P_mphX_-lacZ*)	*	*

a Cells were grown in the presence (+) or absence (−) of 2.0 mM phenol and assayed for β-galactosidase activities as described in Materials and Methods. Values shown are means ± standard deviation of triplicate experiments.

*No detectable activity.

### Binding of MphR to the *mphR-mphK* intergenic region

**Figure 5:**
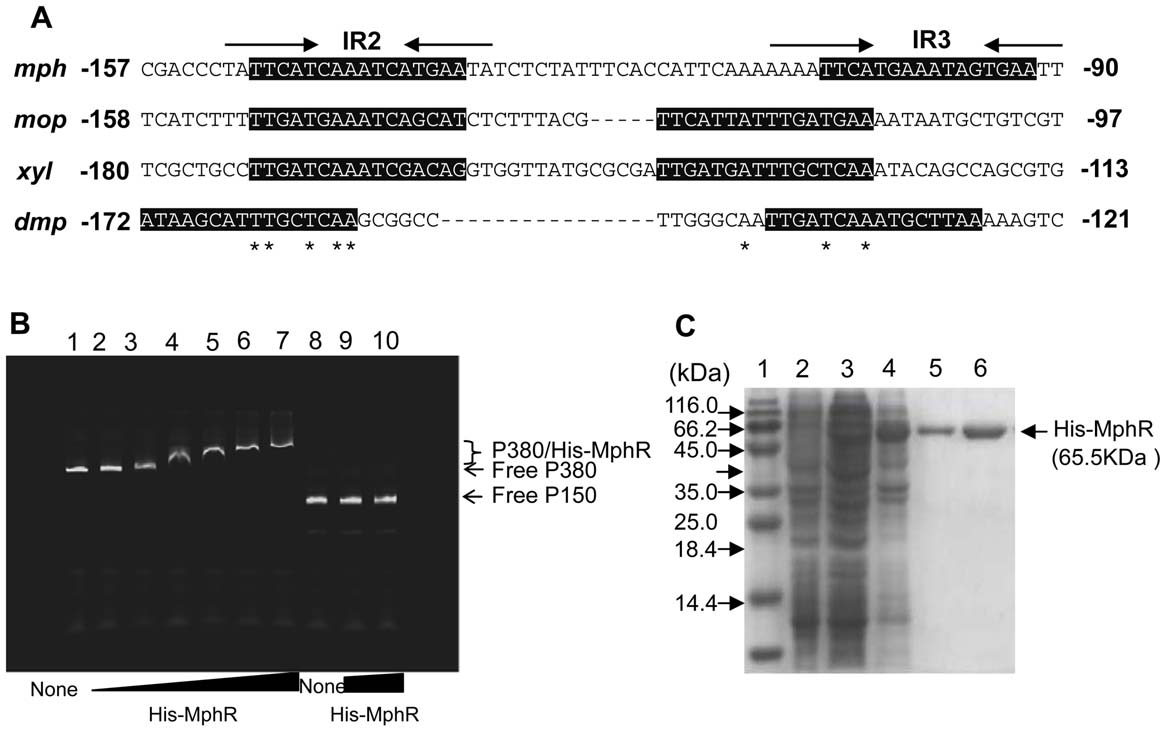
Electrophoretic mobility shift assay. (A) Comparison of the upstream region of the *mphK* promoter with the corresponding regions of the *mop* gene cluster of *A. calcoaceticus* NCIB8250, the *xyl* upper gene cluster of *Pseudomonas putida* mt-2 (TOL), and the *dmp* gene cluster of *Pseudomonas* sp. CF600. Grey-shaded boxes show the putative upstream activating sequences (UASs) in the *mph*, *mop*, *xyl*, and *dmp* gene clusters to which the corresponding XylR/DmpR-type regulators bind. (B) gel retardation analysis of His-MphR binding to the *mphR*-*mphK* intergenic region. Gel-mobility shift assay was performed as described in the text using a 380-bp fragment (P380) covering the *mphR*-*mphK* intergenic region. Lane 1 is a control lane containing 2.5 nM of labeled P380 fragment only. Lane 2–7 contains 2.5 nM of the labeled P380 fragment incubated with 5, 10, 20, 30, 40 and 50 nM His-MphR protein. Lane 8 is containing 2.5 nM of labeled DNA fragment amplified using two primers, IRDye-labeled M13-F and M13-R, and plasmid pGEM T-vector as template, named P150. Lane 9–10 contains 2.5 nM of the labeled P150 fragment incubated with 30 and 50 nM His-MphR protein, respectively. The positions of the free DNA and the MphR-DNA complex are indicated. (C) SDS-PAGE of purified His-MphR protein from *E. coli* BL21 (pEMR). Lane 1: Molecular markers with their masses indicated; Lane 2: Uninduced of BL21 (pEMR); Lane 3: BL21 (pEMR) induced by 0.1 mM IPTG; Lane 4: BL21 (pEMR) induced by 0.5 mM IPTG; Lane 5: MphR purified by NTA resin and eluted by NTA-60; Lane 6: MphR purified by NTA resin and eluted by NTA-80.

As shown in Fig. 5A, a region spanning IR2 and IR3 appears to contain the upstream activating sequences (UASs) of *P_mphK_* because it is similar to those found in the promoter regions of the *mopKLMNOP*, *dmpKLMNOP*, and *xyl* upper pathway gene clusters [22]. In order to investigate the direct interaction of MphR with the intergenic region between *mphR* and *mphK*, we designed a DNA fragment P380 completely covering the *mphK* promoter region from positions −326 to +49 relative to the transcription start site (Fig. 4A). The fragment was mixed with the purified His-MphR (Fig. 5C). Then, the mixtures of DNA/MphR complex and free DNA were resolved by native PAGE. The purified His-MphR protein with P380 caused a band shift of P380. As the amount of protein increased, shifted band was detected, indicating that the MphR protein specifically retards DNA fragments containing the *mphR*-*mphK* promoter region (Fig. 5B). This observation provides evidence for a direct interaction of MphR with the *mphR*-*mphK* promoter region, suggesting an activation mechanism similar to that proposed for a well-studied XylR/DmpR-type regulator MopR in the phenol-degrading bacterium *A. calcoaceticus* NCIB8250 [22].

## Discussion

The expression of phenol catabolic pathway is tightly regulated by one or more regulatory proteins. One of the most extensively studied activators of phenol degradation is the plasmid-borne DmpR from *Pseudomonas* sp. Strain CF600 [17]. DmpR-type regulators consist of four functional domains: the N-terminal signal reception domain (A-domain), the central activation domain with ATPase activity (C-domain), the C-terminal DNA-binding domain with a helix-turn-helix (HTH) motif (D-domain), and a short flexible linker domain between the A-domain and the C-domain (B-domain) [27], [38]. Multiple alignment of MphR with XylR/DmpR-type regulators showed that MphR has an NTP-binding motif (GxxGxGKExxAxxxHxxS, where x is any amino acid, aa 272–aa 289) [15], [39] and an AAA+ protein family signature (GAYTGA, aa 319–aa 324) [40] with one amino acid difference (the complete motif is GAFTGA) in the C-domain. The former is involved in the ATP binding and hydrolysis on the C-domain, while the latter is expected to interact with σ^54^-RNAP. Recently, the molecular mechanism of ATP hydrolysis-mediated transcriptional activation was discussed based on the structures of bacterial enhancer-binding proteins PspF and NtrC by Rappas *et al.* [41]. These proteins have several common amino acid residues important in ATP binding and hydrolysis (K42, E43, N64, D107, E108, and R227 in PspF) and they are all conserved in MphR as K278, E279, N300, D343, E344, and R462, respectively. In the B-domain of MphR, a heptad-repeats signature-like sequence with regularly spaced hydrophobic amino acids is present (MxxxLxxLxxxLxxLxxxIxxxSxxY, aa 213–aa 239), which is involved in dimer formation and prevention of ATP hydrolysis without effector binding [42]. Moreover, the HMMPFAM motif search program (http://hmmpfam.ddbj.nig.ac.jp/top-e.html) predicted the presence of a HTH DNA binding motif in the D-domain (aa 514–aa 555) of MphR. Conservation of these functionally important motifs demonstrates that MphR has many structural and mechanistic features common in NtrC family regulators including XylR/DmpR subclass members and the mechanism for the transcriptional activation by MphR is essentially identical to the above-mentioned mechanism by XylR/DmpR-type regulators.

Although some lines of experimental evidence confirming the repressor function of MphX have been obtained in this study, MphX shows little similarity with the members of known bacterial regulator families. BLAST (http://blast.ddbj.nig.ac.jp/top-e.html) and CDART (http://www.ncbi.nlm.nih.gov/Structure/lexington/lexington.cgi) searches suggest that MphX belongs to the COG4313 family in the COG (Clusters of Orthologous Group) database [43]. Although most members of the COG4313 family are annotated as hypothetical or putative proteins, several proteins are designated as “involved in meta-pathway of phenol degradation”, including those encoded by *orfX* (identity with the encoded protein: 27.8%) in the phenol degradation gene cluster of *C. testosteroni* TA441 [34], *orf1* (27.0%) in the aniline degradation gene cluster of *P. putida* UCC22 [44], and *orfU* (26.3%) in the aniline degradation gene cluster of *D. tsuruhatensis* AD9 [35]. In the phenol degradation gene cluster of TA441 (*aphSRKLMNOPQBorfXYaphTCEFGHJI*), *orfX* is located downstream of a catechol 2,3-dioxygenase gene (*aphB*) [34]. Arai *et al.* [34] pointed out that the *orfX* gene product (OrfX) has a typical amino-terminal signal sequence for membrane translocation, suggesting that OrfX is a protein in the periplasm. Sequence analysis by SignalIP 3.0 (http://www.cbs.dtu.dk/services/SignalP/) predicted that MphX also has a signal peptide sequence at its amino-terminus (aa 1–aa 23, signal peptide probability = 1.000) and its most likely cleavage site (A21F22A23-T24E25, maximum cleavage site probability = 0.999). Thus, MphX has at least one feature specific to periplasmic proteins, but at present the actual location of the MphX protein in PHEA-2 is not known. The same authors found that an *orfXY* mutant of TA441 grew poorly on phenol and accumulated 2-hydroxymuconic semialdehyde [34]. To our knowledge, this is the only description of an MphX homolog-coded gene function. The mutant phenotype might be caused by a mutation in *orfX*, but there are also other possibilities, *i.e.* a mutation in *orfY* or a polar effect for the expression of the downstream *meta*-cleavage pathway genes. In the aniline degradation gene clusters of *D. tsuruhatensis* AD9 and *P. putida* UCC22, MphX homolog-coded genes, *orfU* and *orf1*, are also located between two sets of genes encoding a ferredoxin-like protein and a catechol 2,3-dioxygenase (*tdnD1C*-*tdnD2C2* and *tadD1C1*-*tadD2C2*) [35], [44]. However, the functions of these genes remain unknown. Several MphX homolog-coded genes have been found near alcohol dehydrogenase genes, such as *orf1* near the 4-nitrobenzoate degradation gene cluster of *Pseudomonas* sp. TW3 [45], *qbdB* in the short-chain alcohol degradation gene cluster of *P. putida* HK5 [46], and *chnX* in the cyclohexanol degradation gene cluster of *Acinetobacter* sp. SE19 [47]. These gene products showed 21–22% aa sequence identity with MphX. However, the functions of these genes have not been elucidated yet. Accordingly, MphX is the only protein in this COG4313 family whose function has been demonstrated as a repressor. We looked for DNA binding motifs in MphX using several motif search programs (*e.g.*, http://motif.genome.jp/), but we were unable to detect any HTH motif or other motifs specific to regulatory proteins in MphX.

*Comamonas testosteroni* strains TA441 and R5 have their respective mPH-encoding operon (*aphKLMNOP* and *phcKLMNOP*) and the counterpart XylR/DmpR-type regulatory gene (*aphR* and *phcR*) is in almost the same gene arrangement as that of PHEA-2 [29], [34]. However, strain TA441 has an additional regulatory gene, *aphS*, just downstream of *aphR*, whereas strain R5 has two additional regulatory genes, *phcS* and *phcT*, downstream of *phcR*. MphX shows little sequence similarity with these regulatory proteins. In strain TA441, *aphS* completely represses the transcriptional activation of *aphKLMNOP* caused by *aphR* in the presence of phenol. Consequently, strain TA441 is unable to grow on phenol, but its *aphS*-deficient mutant is able to grow on phenol due to the de-repression [7]. Interestingly, it was found that the transcription of *aphKLMNOP* is also caused by a gratuitous inducer, acetate, and that *aphS* represses this gratuitous activation, suggesting that *aphS* prevents wasteful expression of *aphKLMNOP* induced by such a ubiquitous carbon source [7]. In strain R5, *phcS* also represses the transcriptional activation of *phcKLMNOP* caused by *phcR* in the presence of phenol and similar gratuitous expression by acetate [29]. However, the *phc* gene cluster contains another regulatory gene, *phcT*, and the presence of *phcT* enabled strain R5 to overcome the repression caused by *phcS* and to grow on phenol [28]. The aa sequence analysis of these regulatory gene products indicated that AphS and PhcS belong to the GntR regulator family [7], [29] while PhcT belongs to the AraC/XylS regulator family [28]. One of the AphS binding sites (consensus motif, TATCGTAT) determined by gel-mobility shift assay is located around the putative IHF (Integration Host Factor)-binding site, but not within the putative UASs [7]. Similar binding motifs were also found in the putative IHF-binding site of the *phcR*-*phcK* intergenic region [28]. Hence, it is suggested that these proteins could bind to the putative IHF binding sites and hamper the binding of IHF [15], [28], [48]. However, the IHF-binding consensus motif is absent in the A+T-rich sequence of the *mph* operon of strain PHEA-2. In particular, we were unable to detect any HTH motifs in the MphX protein using several motif search programs (*e.g.*, http://motif.genome.jp/). Moreover, all attempts to perform electrophoretic mobility shift analysis using MphX have failed. So far we have not succeeded in the purification of MphX due to its easily-aggregating property. We presume that the mechanism by which MphX partially represses *mphKLMNOP* transcription differs from those used by AphS and PhcS; however, regulatory mechanism of the *mphX* gene as a repressor requires further investigation.

Most regulatory proteins of aromatic catabolic pathways act as transcriptional activators and regulation by a repressor is generally rare in aromatic compound catabolism [26], [27]. The use of repressors in the regulation of phenol degradation is not yet fully understood. In the case of MphX, the most likely reason is to prevent the wasteful expression of the mPH genes, as previously described for *aphS* [7]. However, we have not yet found such wasteful expression induced by ubiquitous carbon sources in the absence of phenol in PHEA-2. Another reason may be to prevent excess expression of the mPH genes in the presence of phenol. This would result in the accumulation of toxic intermediates, such as catechol and muconate in cells if the expression of downstream pathway genes does not respond to that of the mPH genes. Such accumulation could potentially lead to loss of the ability to degrade aromatic compounds. This has been shown for plasmid-dependent carbazole degradation in *Pseudomonas fluorescens* Pf0-1 where catechol accumulation caused the loss of carbazole-degrading ability [49]. In addition, overproduction of one of the mPH polypeptides (MopN) was reported to cause significant growth inhibition in *Acinetobacter* [50]. However, in the case of PHEA-2, as shown in Fig. 2, the *mphX*-deletion mutant showed faster phenol degradation and better growth on phenol than the wild-type strain, indicating no toxic or inhibitory effects derived from the phenol concentration used. A similar situation was observed even at high concentrations of phenol (up to the degradation limit, 8 mM) (data not shown). Thus, this reason does not seem to be the case. It seems likely that PHEA-2 acquired the *mph* gene cluster through lateral gene transfer from a toxic intermediate (or over-expression)-sensitive strain. Further elucidation of the presence of this repression system and its mechanisms may provide substantial information regarding bacterial survival strategies in natural and polluted environments with phenolic compounds.

## Materials and Methods

### Bacterial strains, plasmids, primers, and growth conditions used

The bacterial strains and plasmids used in this study are listed in Table 2 and the sequences of all the primers used are listed in Table 3. *Acinetobacter* strains were grown at 30°C in mineral salts (MS) medium (NaNO_3_ 0.5 g, K_2_HPO_4_ 0.65 g, KH_2_PO_4_ 0.17 g, MgSO_4_ 0.10 g per liter) supplemented with phenol or sodium lactate, or in Luria-Bertani (LB) medium. *Escherichia coli* strains were grown at 37°C in LB medium. When necessary, ampicillin (20 µg/ml), kanamycin (50 µg/ml), and tetracycline (50 µg/ml) were added to the medium.

**Table 2:** Bacterial strains and plasmids used in this study.

Strain or plasmid	Relevant characteristic(s)^a^	Reference or source
Strains		
*E. coli*		
BL21(DE3)	Carrying a T7 RNA polymerase gene under the control of *lacUV5* promoter	Novagen
JM109	*recA*1 *supE*44 *endA*1 *hsdR*17 *gyrA*96 *relA*1 *thi*Δ(*lac-proAB*) F′[*traD*36 *proAB^+^ lacI^q^ lacZ*ΔM15]	Takara
*A. calcoaceticus*		
PHEA-2	Phenol degrader, Wild type, phenol^+^, Ap^R^	[32]
A2R	*mphR*-deletion mutant of PHEA-2, phenol^−^, Ap^R^	This study
A2N	*mphN*-deletion mutant of PHEA-2, phenol^−^, Ap^R^	This study
A2X	*mphX*-deletion mutant of PHEA-2, phenol^+^, Ap^R^	This study
Plasmids		
pGD926	Tc^R^, RK2-based broad host range, Tra^−^, promoterless *lacZ*,	[52]
pGDP	pGD926 derivative carrying the *P_mphK_*-*lacZ* transcriptional fusion	[53]
pGDRP	pGD926 derivative carrying the *P_mphR_*-*lacZ* transcriptional fusion	This study
pGDPX	pGD926 derivative carrying the *P_mphX_*-*lacZ* transcriptional fusion	This study
pMD18-T	Ap^R^, T-vector for cloning of PCR products	Takala
pGEM-T	Ap^R^, T-vector for cloning of PCR products	Promega
pGE380	Ap^R^, pGEM-T derivative harboring *mphR*-*mphK* intergenic region	This study
pK18mob*sacB*	Km^R^, pK18 derivative harboring *sacB.*	[51]
pMPHKp	pMD18-T derivative carrying a 217-bp fragment from −98 to +119 (relative to the transcriptional start for *mphK*) containing the flanking region and the 5′- end region of *mphK*.	This study

a phenol^+^, growth on phenol; phenol^−^, no growth on phenol; Ap^R^, ampicillin resistance; Tc^R^, tetracycline resistance; Km^R^, kanamycin resistance.

**Table 3:** Primers used in this study.

Name	Sequence (5′-3′) a	Purpose
P*_R_*-F	AA***GGATCC***TTTAACCCAGCTTGATAACC	Amplification of *mphR* promoter region
P*_R_*-R	CGAATTCG***GGATCC***TCTGCATTTAAGTC	
P*_X_*-F	GT***AAGCTTGGAGATAGTCGCACG***	Amplification of *mphX* promoter region
P*_X_*-R	AC***GGATCC***ACACCACATATGATTG	
R1-F	GC***GAATTC***AAGTCTTCAACATTAAC	Construction of *mphR*–deletion mutant
R1-R	AA***GTCGAC***TTTGAGCAATTGTC	
R2-F	TGCGTATCGTCCCGTTGTA	
R2-R	TCGGCTGTGTCCTGTAAAC	
N1-F	AT***GAATTC***GCCACGTTGAACAAAC	Construction of *mphN*–deletion mutant
N1-R	TT***GTCGAC***GATTGATCATCCTTAAATC	
N2-F	AT***GTCGAC***AAGCATGGCTACCTG	
N2-R	TT***GGATCC***AGCTTGGAATTCGATTG	
X1-F	GCAGCGACTACTTATGTGC	Construction of *mphX*–deletion mutant
X1-R	ATTTGGAAGGCAGAACTCCT	
X2-F	AT***GTCGAC***TATAGCTTGGGTTATC	
X2-R	TT***GGATCC***GTGAATTGATGAAGATG	
MphR188-F	TCGAGCCATGAGTGATCTGTT	Amplification of the 380-bp *mphR*-*mphK* intergenic region
MphK207-R	GTACTCCATCAAGACATGGTC	
M13-fwd	CACGACGTTGTAAAACGAC	Amplification of the 380-bp *mphR*-*mphK* intergenic region
M13-rev	GGATAACAATTTCACACAGG	

a Restriction sites for BamHI (5′-GGATCC-3′), HindIII (5′-AAGCTT-3′), EcoRI (5′-GAATTC-3′), and SalI (5′-GTCGAC-3′) are shown in bold and italic.

### Construction of *mphR-*, *mphN-* and *mphX-*deletion mutants

In order to disrupt key genes in phenol degradation, the upstream and downstream regions of *mphR*, *mphN*, and *mphX* were generated by PCR using the total DNA of PHEA-2 as the template and several primer sets shown in Table 3. The amplified PCR products were purified and ligated into the pMD18-T vector (Takara). The resulting plasmids were double-digested with EcoRI and SalI (for plasmids carrying the upstream region) or SalI and BamHI (for those carrying the downstream region) and purified. The upstream and downstream fragments for each gene were cloned together into the EcoRI/BamHI sites of pK18mobsacB [51]. The resulting plasmids were introduced into *E. coli* JM109 and then transferred into PHEA-2 by triparental mating [52]. Integration of the introduced plasmid into the chromosome by the first crossover was selected on LB plates containing kanamycin (25 µg/ml) and ampicillin (20 µg/ml). The second crossover cells were selected by culture on LB plates containing 10% (w/v) sucrose and ampicillin (20 µg/ml). The selected cells were analyzed by PCR to confirm that gene replacement had occurred (data not shown). The resulting *mphR*, *mphN* and *mphX* deletion mutants were designated as A2R, A2N and A2X, respectively.

### Phenol degradation test

*Acinetobacter* strains were grown overnight in LB medium and the cultures were harvested by centrifugation (8,000×g, 4°C, 5 min). The cells were washed twice with MS medium and suspended at an OD_600_ of 0.15 with MS medium containing 2 mM phenol. Cell suspensions were further incubated with shaking at 200 rpm on a rotary shaker. Aliquots of the cultures were sampled at specific intervals and centrifuged (13,000×*g*, 4°C, 1 min) to remove the cells. Following cell removal, phenol concentrations in the supernatants were determined using the 4-aminoantipyrine colorimetric method. This analysis was performed according to the procedures described in Folsom *et al.* [1].

### Assay for phenol-oxygenating activity

*Acinetobacter* strains were grown in LB medium to the late-log phase and the cultures were harvested by centrifugation (8,000×*g*, 4°C, 5 min). The cells were washed twice with MS medium, suspended at an OD_600_ of 0.7 with MS medium containing 2 mM phenol or 5 mM sodium lactate and further incubated at 30°C for 6 h for induction. Then, the cells were again collected by centrifugation, washed with MS medium, and resuspended at an OD_600_ of 1.5 in 40 ml potassium phosphate buffer (100 mM, pH 7.5). After measurement of endogenous oxygen uptake for 5 min, reactions were started by adding phenol to the cultures to a final concentration of 0.1 mM. Oxygen concentrations in the cultures were monitored at 25°C for 3 min using a YSI 5000 Clark-type oxygen electrode (Yellow Springs Instruments, Yellow Springs, USA) and oxygen uptake rates were corrected for endogenous respiration. Phenol-oxygenating activity was expressed as µmoles of O_2_ uptake per min per gram of dry cell. The final data were obtained from three independent experiments and shown as mean ± standard error.

### Construction of plasmids containing *mphR*, *mphN and mphX* promoter-*lacZ* transcriptional fusion

The construction of *mphR* or *mphX* promoter-lacZ transcriptional fusion was similar to that of *P_mphK_*-*lacZ* fusion [33], [53]. The flanking region and the 5′-end region of the *mphR* and *mphX* genes were generated by PCR using the total DNA of PHEA-2 as the template and primer sets shown in Table 3. PCR products were purified and ligated into the pGEM-T vector (Promega, Madison, USA). The resulting plasmids were double-digested with BamHI and HindIII. The digested fragments were ligated into the BamHI/HindIII site of a RK2-based broad host range vector, pGD926, with a promoterless *lacZ* gene [52] in order to develop promoter-*lacZ* fusions and to introduce them into *Acinetobacter* strains. The resulting plasmids with the *P_mphR_*- or *P_mphX_*-*lacZ* fusion were designated as pGDRP and pGDPX, respectively.

### β-Galactosidase assay

For the measurement of β-galactosidase activity in *Acinetobacter* strains, overnight cultures were washed twice with MS medium and suspended at an OD_600_ of 0.2 with MS medium containing 5 mM sodium lactate with and without 2 mM phenol and further incubated for 6 h with shaking at 200 rpm on a rotary shaker for induction. β-Galactosidase activity of cell suspensions prepared from three independently grown cultures was measured as described by Miller [54] and the values obtained were averaged and expressed in Miller units (U).

### Primer extension analysis

In order to map the transcriptional start site for *mphK*, the total RNA of PHEA-2 was isolated from mid-log phase cells grown in LB medium containing 1 mM phenol using a RNAspin Mini kit (GE Healthcare Bio-Sciences, Tokyo, Japan) according to the supplier's instructions. For primer extension, an oligonucleotide labeled with FAM at its 5′ end (named Pk-FAM, 5′-CTCTGCATTTAAGTCGCCAGTG-3′) was custom-synthesized by and purchased from Gene Design Inc. (Osaka, Japan). Annealing and primer extension were performed using a Quanti Tect Reverse Transcription Kit (Qiagen, Tokyo, Japan) according to the supplier's instructions. Detection and sequencing of the DNA fragments extended from the FAM-labeled primer were carried out using a DSQ-2000L autosequencer (Shimadzu, Kyoto, Japan) and a 8% (v/v) polyacrylamide (Long Ranger, Takara) gel. The sequencing reaction was carried out using a Thermo Sequenase Labeled Primer Cycle Sequencing Kit with 7-deaza-dGTP (GE Healthcare Bio-Sciences) according to the manufacture's instructions. The pMPHKp plasmid (Table 2), which has a 217-bp insert fragment spanning the flanking region and the 5′-end region of *mphK* (from −98 to +119), was used as the template for sequencing.

### Purification of histidine-tagged MphR

The full-length *mphR* gene was ligated into the pET-28 vector [53] and transformed into *E. coli* BL21 (DE3) to express the His-tagged MphR. The transformants were cultured in LB medium containing kanamycin and induced with 0.5 mM isopropyl-β-D-thiogalactopyranoside (IPTG) for 16 h at 10°C. The cells were harvested by centrifugation and disrupted by sonication (160W, 2 s bursts with a 3 s interval between every two bursts) for 10 min on ice. The lysate was centrifuged (20,000×*g*, 4°C, 20 min) and the supernatant was immediately used for protein purification by the nickel-nitrilotriacetic acid (Ni-NTA) columns (Qiagen) according to the manufacture's recommendations. Protein concentrations were measured by the Bradford method [55].

### Gel mobility-shift assay

For the gel mobility-shift assay, a fragment (380 bp) containing the intact *mphR-mphK* intergenic region was prepared (Fig. 4A). First, the intergenic region was amplified by PCR using two primers, MphR188-F and MphK207-R (Table 3), and PHEA-2 total DNA as the template. Then, the amplified fragment was purified and ligated into the pGEM-T vector (Promega). The resulting construct was designated as pGE380 (Table 2). The sequence of the insert fragment was confirmed by sequencing. Secondly, the intergenic region was amplified again using a different primer set (M13-fwd and M13-rev) labeled with IRD800 infrared dye at the 5′ end (Table 3) (Li-cor) and pGE380 as the template. At the same time, the control DNA fragment was amplified using the same primer set and the pGEM-T vector as the template. The amplified fragment was electrophoretically purified from a 2% (w/v) low temperature-melting agarose gel (Cambrex, East Rutherford, USA) according to the protocol and named P380. Binding reaction mixtures contained 20 mM Tris-HCl (pH 7.5), 50 mM KCl, 6 mM DTT, 0.5% (v/v) Tween-20, 0.05 µg/µl poly (dI-dC) (Roche, Mannheim, Germany), 2.5 nM DNA fragment and the purified histidine-tagged (His-) MphR with the increasing concentrations in a final volume of 10 µl. After incubation for 20 min at 30°C in darkness, 1 µl of 10× Orange loading dye (0.02 g orange G, 6 ml 50% [v/v] glycerol, 1.2 ml 0.5 M EDTA [pH 8.0], 2.87 ml sterile water) was added to the mixtures, mixed briefly, and then loaded onto a 8% polyacrylamide gel. Electrophoresis was carried out at 10 V/cm for about 3 h in 0.5× TBE buffer (45 mM Tris borate, 1 mM EDTA) in darkness. Scanning of the gel was conducted using an Odyssey Imager (LI-COR) under the supplier's recommended condition.

## References

[1] Folsom BR, Chapman PJ, Pritchard PH (1990). Phenol and trichloroethylene degradation by *Pseudomonas cepacia* G4: kinetics and interactions between substrates.. Appl Environ Microbiol.

[2] Heinaru E, Truu J, Stottmeister U, Heinaru A (2000). Three types of phenol and p-cresol catabolism in phenol- and p-cresol-degrading bacteria isolated from river water continuously polluted with phenolic compounds.. FEMS Microbiol Ecol.

[3] Kalin M, Neujahr HY, Weissmahr RN, Sejlitz T, Johl R (1992). Phenol hydroxylase from Trichosporon cutaneum: gene cloning, sequence analysis, and functional expression in *Escherichia coli*.. J Bacteriol.

[4] Kukor JJ, Olsen RH (1992). Complete nucleotide sequence of tbuD, the gene encoding phenol/cresol hydroxylase from *Pseudomonas pickettii* PKO1, and functional analysis of the encoded enzyme.. J Bacteriol.

[5] Duffner FM, Kirchner U, Bauer MP, Muller R (2000). Phenol/cresol degradation by the thermophilic *Bacillus thermoglucosidasius* A7: cloning and sequence analysis of five genes involved in the pathway.. Gene.

[6] Omokoko B, Jantges UK, Zimmermann M, Reiss M, Hartmeier W (2008). Isolation of the phe-operon from *G. stearothermophilus* comprising the phenol degradative meta-pathway genes and a novel transcriptional regulator.. BMC Microbiol.

[7] Arai H, Akahira S, Ohishi T, Kudo T (1999). Adaptation of *Comamonas testosteroni* TA441 to utilization of phenol by spontaneous mutation of the gene for a trans-acting factor.. Mol Microbiol.

[8] Ehrt S, Schirmer F, Hillen W (1995). Genetic organization, nucleotide sequence and regulation of expression of genes encoding phenol hydroxylase and catechol 1,2-dioxygenase in *Acinetobacter calcoaceticus* NCIB8250.. Mol Microbiol.

[9] Nordlund I, Powlowski J, Shingler V (1990). Complete nucleotide sequence and polypeptide analysis of multicomponent phenol hydroxylase from *Pseudomonas* sp. strain CF600.. J Bacteriol.

[10] Sandhu A, Halverson LJ, Beattie GA (2009). Identification and genetic characterization of phenol-degrading bacteria from leaf microbial communities.. Microb Ecol.

[11] Li D, Yan Y, Ping S, Chen M, Zhang W (2010). Genome-wide investigation and functional characterization of the beta-ketoadipate pathway in the nitrogen-fixing and root-associated bacterium *Pseudomonas stutzeri* A1501.. BMC Microbiol.

[12] O'Neill E, Wikstrom P, Shingler V (2001). An active role for a structured B-linker in effector control of the sigma54-dependent regulator DmpR.. EMBO J.

[13] Park SM, Park HH, Lim WK, Shin HJ (2003). A new variant activator involved in the degradation of phenolic compounds from a strain of *Pseudomonas putida*.. J Biotechnol.

[14] Shingler V (2003). Integrated regulation in response to aromatic compounds: from signal sensing to attractive behaviour.. Environ Microbiol.

[15] Sze CC, Laurie AD, Shingler V (2001). In vivo and in vitro effects of integration host factor at the DmpR-regulated sigma(54)-dependent Po promoter.. J Bacteriol.

[16] Butler JE, He Q, Nevin KP, He Z, Zhou J (2007). Genomic and microarray analysis of aromatics degradation in *Geobacter metallireducens* and comparison to a Geobacter isolate from a contaminated field site.. BMC Genomics.

[17] Sarand I, Skarfstad E, Forsman M, Romantschuk M, Shingler V (2001). Role of the DmpR-mediated regulatory circuit in bacterial biodegradation properties in methylphenol-amended soils.. Appl Environ Microbiol.

[18] Powlowski J, Shingler V (1994). Genetics and biochemistry of phenol degradation by *Pseudomonas* sp. CF600.. Biodegradation.

[19] Shingler V, Bartilson M, Moore T (1993). Cloning and nucleotide sequence of the gene encoding the positive regulator (DmpR) of the phenol catabolic pathway encoded by pVI150 and identification of DmpR as a member of the NtrC family of transcriptional activators.. J Bacteriol.

[20] Muller C, Petruschka L, Cuypers H, Burchhardt G, Herrmann H (1996). Carbon catabolite repression of phenol degradation in *Pseudomonas putida* is mediated by the inhibition of the activator protein PhlR.. J Bacteriol.

[21] Ng LC, Poh CL, Shingler V (1995). Aromatic effector activation of the NtrC-like transcriptional regulator PhhR limits the catabolic potential of the (methyl)phenol degradative pathway it controls.. J Bacteriol.

[22] Schirmer F, Ehrt S, Hillen W (1997). Expression, inducer spectrum, domain structure, and function of MopR, the regulator of phenol degradation in *Acinetobacter calcoaceticus* NCIB8250.. J Bacteriol.

[23] Teramoto M, Futamata H, Harayama S, Watanabe K (1999). Characterization of a high-affinity phenol hydroxylase from Comamonas testosteroni R5 by gene cloning, and expression in *Pseudomonas aeruginosa* PAO1c.. Mol Gen Genet.

[24] Cases I, de Lorenzo V (2005). Promoters in the environment: transcriptional regulation in its natural context.. Nat Rev Microbiol.

[25] Ramos JL, Marques S, Timmis KN (1997). Transcriptional control of the *Pseudomonas* TOL plasmid catabolic operons is achieved through an interplay of host factors and plasmid-encoded regulators.. Annu Rev Microbiol.

[26] Diaz E, Prieto MA (2000). Bacterial promoters triggering biodegradation of aromatic pollutants.. Curr Opin Biotechnol.

[27] Tropel D, van der Meer JR (2004). Bacterial transcriptional regulators for degradation pathways of aromatic compounds.. Microbiol Mol Biol Rev.

[28] Teramoto M, Ohnishi K, Harayama S, Watanabe K (2002). An AraC/XylS family member at a high level in a hierarchy of regulators for phenol-metabolizing enzymes in *Comamonas testosteroni* R5.. J Bacteriol.

[29] Teramoto M, Harayama S, Watanabe K (2001). PhcS represses gratuitous expression of phenol-metabolizing enzymes in *Comamonas testosteroni* R5.. J Bacteriol.

[30] Laurie AD, Bernardo LM, Sze CC, Skarfstad E, Szalewska-Palasz A (2003). The role of the alarmone (p)ppGpp in sigma N competition for core RNA polymerase.. J Biol Chem.

[31] Petruschka L, Burchhardt G, Muller C, Weihe C, Herrmann H (2001). The cyo operon of *Pseudomonas putida* is involved in carbon catabolite repression of phenol degradation.. Mol Genet Genomics.

[32] Xu Y, Chen M, Zhang W, Lin M (2003). Genetic organization of genes encoding phenol hydroxylase, benzoate 1,2-dioxygenase alpha subunit and its regulatory proteins in *Acinetobacter calcoaceticus* PHEA-2.. Curr Microbiol.

[33] Zhan Y, Yu H, Yan Y, Chen M, Lu W (2008). Genes involved in the benzoate catabolic pathway in *Acinetobacter calcoaceticus* PHEA-2.. Curr Microbiol.

[34] Arai H, Ohishi T, Chang MY, Kudo T (2000). Arrangement and regulation of the genes for meta-pathway enzymes required for degradation of phenol in *Comamonas testosteroni* TA441.. Microbiology.

[35] Liang Q, Takeo M, Chen M, Zhang W, Xu Y (2005). Chromosome-encoded gene cluster for the metabolic pathway that converts aniline to TCA-cycle intermediates in *Delftia tsuruhatensis* AD9.. Microbiology.

[36] Peng Z, Yan Y, Xu Y, Takeo M, Yu H (2010). Improvement of an *E. coli* bioreporter for monitoring trace amounts of phenol by deletion of the inducible sigma54-dependent promoter.. Biotechnol Lett.

[37] Wigneshweraraj SR, Burrows PC, Bordes P, Schumacher J, Rappas M (2005). The second paradigm for activation of transcription.. Prog Nucleic Acid Res Mol Biol.

[38] Ng LC, O'Neill E, Shingler V (1996). Genetic evidence for interdomain regulation of the phenol-responsive final sigma54-dependent activator DmpR.. J Biol Chem.

[39] Morett E, Segovia L (1993). The sigma 54 bacterial enhancer-binding protein family: mechanism of action and phylogenetic relationship of their functional domains.. J Bacteriol.

[40] Dago AE, Wigneshweraraj SR, Buck M, Morett E (2007). A role for the conserved GAFTGA motif of AAA+ transcription activators in sensing promoter DNA conformation.. J Biol Chem.

[41] Rappas M, Bose D, Zhang X (2007). Bacterial enhancer-binding proteins: unlocking sigma54-dependent gene transcription.. Curr Opin Struct Biol.

[42] Garmendia J, de Lorenzo V (2000). The role of the interdomain B linker in the activation of the XylR protein of *Pseudomonas putida*.. Mol Microbiol.

[43] Tatusov RL, Galperin MY, Natale DA, Koonin EV (2000). The COG database: a tool for genome-scale analysis of protein functions and evolution.. Nucleic Acids Res.

[44] Fukumori F, Saint CP (2001). Complete nucleotide sequence of the catechol metabolic region of plasmid pTDN1.. J Gen Appl Microbiol.

[45] James KD, Hughes MA, Williams PA (2000). Cloning and expression of ntnD, encoding a novel NAD(P)(+)-independent 4-nitrobenzyl alcohol dehydrogenase from *Pseudomonas* sp. Strain TW3.. J Bacteriol.

[46] Toyama H, Fujii T, Aoki N, Matsushita K, Adachi O (2003). Molecular cloning of quinohemoprotein alcohol dehydrogenase, ADH IIB, from *Pseudomonas putida* HK5.. Biosci Biotechnol Biochem.

[47] Cheng Q, Thomas SM, Kostichka K, Valentine JR, Nagarajan V (2000). Genetic analysis of a gene cluster for cyclohexanol oxidation in *Acinetobacter* sp. Strain SE19 by in vitro transposition.. J Bacteriol.

[48] Hales LM, Gumport RI, Gardner JF (1994). Determining the DNA sequence elements required for binding integration host factor to two different target sites.. J Bacteriol.

[49] Takahashi Y, Shintani M, Li L, Yamane H, Nojiri H (2009). Carbazole-degradative IncP-7 plasmid pCAR1.2 is structurally unstable in *Pseudomonas fluorescens* Pf0-1, which accumulates catechol, the intermediate of the carbazole degradation pathway.. Appl Environ Microbiol.

[50] Schirmer F, Hillen W (1998). The *Acinetobacter calcoaceticus* NCIB8250 mop operon mRNA is differentially degraded, resulting in a higher level of the 3′ CatA-encoding segment than of the 5′ phenolhydroxylase-encoding portion.. Mol Gen Genet.

[51] Schafer A, Tauch A, Jager W, Kalinowski J, Thierbach G (1994). Small mobilizable multi-purpose cloning vectors derived from the *Escherichia coli* plasmids pK18 and pK19: selection of defined deletions in the chromosome of *Corynebacterium glutamicum*.. Gene.

[52] Ditta G, Schmidhauser T, Yakobson E, Lu P, Liang XW (1985). Plasmids related to the broad host range vector, pRK290, useful for gene cloning and for monitoring gene expression.. Plasmid.

[53] Zhan Y, Yu H, Yan Y, Ping S, Lu W (2009). Benzoate catabolite repression of the phenol degradation in *Acinetobacter calcoaceticus* PHEA-2.. Curr Microbiol.

[54] Miller JH (1972). Experiments in molecular genetics.

[55] Bradford MM (1976). A rapid and sensitive method for the quantitation of microgram quantities of protein utilizing the principle of protein-dye binding.. Anal Biochem.

